# Effect of a Bio-Based Dispersing Aid (Einar^®^ 101) on PLA-Arbocel^®^ Biocomposites: Evaluation of the Interfacial Shear Stress on the Final Mechanical Properties

**DOI:** 10.3390/biom10111549

**Published:** 2020-11-13

**Authors:** Laura Aliotta, Vito Gigante, Patrizia Cinelli, Maria-Beatrice Coltelli, Andrea Lazzeri

**Affiliations:** 1 Department of Civil and Industrial Engineering, University of Pisa, Via Diotisalvi, 2, 56122 Pisa, Italy; laura.aliotta@dici.unipi.it (L.A.); vito.gigante@dici.unipi.it (V.G.); patrizia.cinelli@unipi.it (P.C.); maria.beatrice.coltelli@unipi.it (M.-B.C.); 2 Interuniversity National Consortium of Materials Science and Technology (INSTM), Via Giusti 9, 50121 Florence, Italy; 3 Planet Bioplastics s.r.l., Via San Giovanni Bosco 23, 56127 Pisa, Italy

**Keywords:** biocomposites, natural fibers, poly (lactic) acid (PLA), interfacial shear strength, mechanical properties

## Abstract

In this paper, the production and the characterization of poly (lactic) acid (PLA)-based composites containing different amounts (from 10 wt.% to 25 wt.%) of ultra-short cellulose fibers (Arbocel 600 BE/PU) have been investigated. On the basis of a previous study, it was observed that the addition of the cellulose fibers led to an embrittlement of the composite. Consequently, in order to obtain a composite with enhanced impact resistance and elongation at break, the effect of the Einar 101 addition (a bio-based dispersing aid additive) was analyzed. The role of the adhesion between the fiber and the matrix, coupled with a better fiber dispersion, was thus evaluated. Also, the consequences on the final mechanical properties (tensile and impact test) caused by the Einar addition were investigated. Analytical models were also applied in order to obtain an evaluation of the variation of the interfacial shear stress (IFSS) (strictly correlated to the fiber-matrix adhesion) caused by the Einar introduction. Furthermore, due to the very low aspect ratio of the Arbocel fibers, a suitable Bader and Boyer model variation was adopted in order to have a better quantitative estimation of the IFSS value.

## 1. Introduction

In this century, remarkable steps forward have been made in the field of “green polymer science”. The driving force is represented by biocomposites that are composite materials obtained by coupling natural fibers into a bio-based or biodegradable matrix. Biopolymers have allowed to overcome many problems related to the impossibility of using biopolymers alone in different sectors [1,2]. For example, natural biofiber composites can be a feasible alternative to classical glass fibers, for those applications in which the control of the final density is of primary interest (for example, in automotive or buildings) [3]. Furthermore, in sectors like single-use packaging or horticulture, the use of entirely biodegradable biopolymer is an enormous advantage that will lead to an expected increase of the demand of this class of material [4,5]. Biocomposites are not only an ecological, but also a low-cost alternative, especially were the natural fibers derive from industrial and/or agricultural wastes and thus they can be valorized [6,7]. It is therefore evident how the investigation of different biocomposites systems is of actual interest, as demonstrated by different reviews present in the literature [8,9,10,11,12]. 

Among the biodegradable matrices available on the market, poly (lactic) acid (PLA) is the most attractive thanks to its biodegradability, renewability, mechanical properties (Young modulus of about 3 GPa, tensile strength between 50 and 70 MPa, elongation at break around 3–6% and impact strength close to 2.5–3 kJ/m^2^ [13,14]) and low production cost if compared to other biodegradable materials [15,16,17]. These features make PLA adaptable in many areas of Engineering and Technology [18,19,20,21]. However, there are some PLA drawbacks (such as low flexibility, excessive brittleness, low thermal stability, low crystallization rates) that highly restrict its possible applications [22,23].

Thanks to the composites’ ability to show superior thermo-mechanical properties compared to their individual components, some of the PLA drawbacks before mentioned can be overcome. These composites’ features coupled with the current requirement to intensify the use of natural fibers with low added value combined with the easy processability and economy of the final biocomposite material has led to the growth of short-fiber reinforced polymer (SFRP) composites [24,25,26]. 

SFRP composites fill the gap between the mechanical properties of continuous fiber composites and unreinforced polymer matrices having poor load-bearing capacity [27]. In some cases, especially when the fibers are very short and randomly oriented, the resulting composites do not necessarily provide enhanced mechanical properties, but in some cases, the benefits obtained by the SFRP composites’ production are related to costs saving, lighter weight and better degradability [22,28].

Different natural fibers and particulate fillers for polymeric matrices have been investigated in the literature, such as mussel shells, flax, hemp, kenaf, jute, ramie, banana, artichoke, cellulose fibers, etc. [29,30,31,32,33,34,35,36]. However, natural fibers containing a large amount of cellulose, hemicellulose and lignin tend to have weak interfacial bonding with the matrix due to their polar hydrophilic tendency that contrasts the polar and hydrophobic properties of the polymer matrices. So, different strategies were investigated and can be found in the literature to overcome these deficiencies in compatibility and interfacial bond strength [37,38,39,40,41]. 

The characterization of fiber/matrix adhesion and the interpretation of mechanical properties is hence fundamental; in particular, it is of primary interest to understand how the complex interactions between short fibers and the surrounding matrix influence the composite final mechanical behavior; for this purpose, different attempts can be found in the literature [42,43,44,45,46]. The mechanical behavior of SFRP composites depends on the efficiency of the stress transferring from the matrix to the fibers, which in turns depends on the fiber and matrix strengths, fibers distribution and the strength of interfacial adhesion [47,48,49]. Generally, the capacity of transferring the load-bearing capacity from the matrix to the fiber is expressed in terms of interfacial shear strength (IFSS or τ). In turn, this parameter is correlated to different factors like the interface thickness, the adhesion strength, the surface energy filler, etc. 

If, from one side, a good adhesion (high IFSS) is responsible for the strength at break increment; on the other hand, a toughness improvement (increased elongation at break and impact resistance at the expense of stress at break decrement) is achieved where low adhesion (low IFSS value) is obtained. In both cases, however, a good fibers dispersion is fundamental in order to avoid agglomeration phenomena that can act as stress concentration factors, leading to a premature failure of the material [50,51,52]. 

It is therefore evident that depending on the final mechanical properties that must be improved, the evaluation of the interfacial adhesion (IFSS) is fundamental (for example, in the evaluation of the effect of particular additives on the fiber/matrix interactions). 

The experimental evaluation of IFSS is often complicated and laborious and, especially for the case of ultra-short fiber composites, can be impossible if the fiber available is not sufficiently long to perform the experimental characterization. Consequently, analytical models are often applied. For this purpose, the Bader and Bowyer (B-B) model (based on the stress-strain curves analysis of the reinforced composite) is mostly adopted. The B-B model, deriving from the Kelly-Tyson equations, assumes a constant shear stress at the fiber-matrix interface due to either the matrix yielding or interfacial debonding [53,54,55]. This well-known model is very intuitive and simple to use; nevertheless, it has been observed that divergences occur when this model is applied for short (aspect ratio < 20) and especially for ultra-short (aspect ratio < 10) fiber composites [24]. In fact, it must be kept in mind that the mechanical response of short-fiber composites differs greatly from long fiber composites, and this difference becomes more and more marked as the fiber aspect ratio (the ratio between the fiber length and its diameter) decreases. The load is transferred from the matrix to the fibers by shear stresses at the fibers’ surfaces (ends effects). These ends effects in the case of long fiber composites are negligible because they involve a small portion of fiber. However, for short and very short fiber composites, these ends effects must be considered [24]. In the case of ultra-short fiber composites, a modified version of the Kelly-Tyson model and consequently of the B-B model must be applied.

On the basis of a previous work [22], a system of PLA containing different amounts (from 10 up to 25 wt.%) of ultra-short cellulosic fibers (Arbocel^®^ 600 BE/PU) was further investigated. In particular, due to the agglomeration tendency of these fibers inside the polymeric matrix, the effect of the addition of a bio-based dispersing aid (Einar^®^ 101) was evaluated. This liquid dispersing aid should coat the fibers, guaranteeing a homogeneous dispersion, and at the same time, should decrease the interaction between polymer matrix and the fibers. In this way, it should be possible to obtain a SFRP composite containing a high quantity of fibers and at the same time, improve the final ductility and impact resistance of the material. The effect of Einar^®^ 101 on this composite system was also evaluated using different existing analytical models. In particular, the Pukánszky [56] model and Sato and Furukawa [57] models were applied. Furthermore, to estimate the IFSS value, the B-B models (the classical version and the modified ones) were adopted, and it has been shown how for the case of ultra-short fiber composites, it is convenient to use the modified approach of the B-B model.

## 2. Materials and Methods

### 2.1. Materials

The materials used for the composites’ preparation are:

1. Poly (lactic) acid (PLA) 2003D, purchased from NatureWorks (Minnesota, Minneapolis, MN, USA), was used. It derives completely from renewable resources and this grade contains about 4% od D-lactic acid units in order to lower the melting point and the crystallization tendency. PLA2003D is a transparent general-purpose extrusion grade biopolymer that can be used naturally or as part of formulated blends or composites. Thanks to its high molecular weight, this PLA grade can be easily processed on conventional extrusion equipment. According to the producer’s data sheet, PLA 2003D has a density of 1.24 g/cm^3^, a melt flow index (MFI) of 6 g/10 min (210 °C, 2.16 kg) and a nominal average molar mass of 200,000 g/mol.
2. Arbocel^®^ 600 BE/PU, provided by J Rettenmaier and Söhne (Rosenberg, Germany), are 100% natural ultra-short, highly pure white micro-cellulose fibers (mean diameter 20 µm, mean fiber length 60 µm, and consequently, mean aspect ratio of 3, bulk density: 200–260 g/L, fiber density 1.44 g/cm^3^). In the following paper, these fibers will be named Arbocel.
3. Einar^®^ 101 provided by Palsgaard (Juelsminde, Denmark), is yellowish viscous and food-grade, entirely based on vegetable oils dispersing aid (density: 1.62g/cm^3^, viscosity: 7 Pa s). It is a Polyglycerol ricinoleate oil with a maximum acid value of 3 mg KOH/g and a saponification value of 170 mg KOH/g. The addition of Einar^®^ 101 should enable a better fiber dispersion and at the same time, should enable to reach higher fiber loads, decreasing the melt viscosity during the processing. In the following paper, this dispersing aid will be named Einar.

### 2.2. Composites’ Preparation

PLA composites containing increasing amounts of Arbocel fibers were produced in pellets. Two series of blends from 10 to 25 wt.% of fiber were obtained: one series with only Arbocel fibers and the other one with the addition of Einar (introduced in a fixed amount of 5 wt.% according to the instructions of the producer’s data sheet). The composites’ names with their composition are reported in Table 1. 

The composites pellets were produced with a semi-industrial Comac EBC 25HT (L/D = 44) (Comac, Cerro Maggiore, Italy) twin screw extruder. Before the extrusion, all solid materials were dried in a Piovan DP 604–615 dryer (Piovan S.p.A., Verona, Italy). PLA granules were introduced into the main extruder feeder, while Arbocel fibers were fed by a specific feeder (suitable for natural fibers), which allows the fixed weight percentage of filler to be added, at a constant concentration in the melt during the extrusion. Einar, being a viscous liquid, was introduced into the extruder by a Verdeflex-Vantage 3000 peristaltic pump (Verder Liquids, Utrecht, Netherland) suitably calibrated to guarantee a constant flow rate. Due to the Einar high viscosity at room temperature, it was heated and pumped into the extruder at a constant temperature of 40 °C. In this way, lowering the Einar viscosity, it was possible to maintain a constant feeding of the liquid into the extruder.

During the extrusion, the temperatures in the zones from 1 to 11 were: 150/175/180/180/180/185/185/185/185/190/190 °C, with the die zone at 190 °C for the composites without Einar. With the Einar addition, the viscosity slows down, and consequently, the entire extruder temperature profile was decreased, and in the zones from 1 to 11, the temperatures were: 140/150/160/160/160/165/165/165/170/170/170 °C, with the die zone at 170 °C. The screw rate was 250 rpm. The extruded strands were cooled in a water bath at room temperature and reduced in pellets by an automatic knife cutter. All pellets were finally dried in a Piovan DP 604–615 dryer at 60 °C.

### 2.3. Melt Flow Rate

A CEAST Melt Flow Tester M20 (Instron, Canton, MA, USA) equipped with an encoder was used to investigate the composites’ melt flow behavior. Thanks to the encoder able to follow the piston movement, it was possible to collect the melt volume rate (MVR) data. The ISO 1133D [58] procedure for the melt flow rate (MFR) evaluation was carried out (190 °C, 2.16 kg). At least five measurements for each composition were made and the mean value was reported.

### 2.4. Specimens Preparation

After the extrusion, the composites pellets were injection-molded using a Megatech H10/18 injection molding machine (TECNICA DUEBI s.r.l., Fabriano, Italy) to obtain dog-bone (ISO 527-1A, width: 10 mm, thickness: 4 mm, length: 80 mm), and parallelepiped Charpy specimens were subsequently V-notched (ISO 179 [59], width: 10 mm, thickness: 4 mm, length: 80 mm, V-notch: 2 mm). The operative conditions of the injection molding process are reported in Table 2.

### 2.5. Mechanical Tests

Tensile tests were carried out, at room temperature, at a crosshead speed of 10 mm/min on an MTS Criterion model 43 universal tensile testing machine (MTS Systems Corporation, Eden Prairie, MN, USA) equipped with a 10 kN load cell and interfaced with a computer running MTS Elite Software. Tests were conducted after 3 days from specimen injection molding, and during these 3 days, the specimens were kept in a dry keeper (SANPLATEC Corp., Osaka, Japan) at controlled atmosphere (room temperature and 50% of humidity). At least ten specimens were tested for each composite composition and the average values were reported. 

Impact tests were performed on V-notched Charpy specimens using a 15 J Instron CEAST 9050 Charpy pendulum (INSTRON, Canton, MA, USA). Also, in this case, the impact tests were carried out after keeping the specimen in the dry keeper for 3 days after the injection molding process. At least ten specimens, at room temperature, were tested, and the average Charpy impact resistance was reported.

### 2.6. Scanning Electron Microscopy Analysis (SEM) 

Composites’ morphology was investigated using cryogenic fractured cross-sections of the Charpy samples. The SEM analysis was carried out, after a platinum sputtering (LEICA EM ACE 600 High Vacuum Sputter Coater, Wetzlar, Germany), on a FEI Quanta 450 FEG scanning electron microscope (Thermo Fisher Scientific, Waltham, MA, USA). In this way, it was possible to observe the fibers’ distribution inside the PLA matrix and it was also possible to have a qualitative idea of the fiber-matrix adhesion.

### 2.7. Thermo-Mechanical Analysis

Dynamic mechanical thermal analysis (DMTA) was carried out using a Gabo Eplexor^®^ DMTA (Gabo Qualimeter, Ahiden, Germany) equipped with a 100 N load cell. At least three specimens were tested for each formulation. Test bars (width: 10 mm, thickness: 4 mm, length: 50 mm) were obtained by cutting the dog-bone tensile specimens. The samples were mounted on the machine in tensile configuration. The used temperature range varied from 20 to 120 °C with heating rate of 1.5 °C/min and at a constant frequency of 1 Hz. 

## 3. Theoretical Analysis

In this work, different analytical models were applied in order to evaluate the effect of the Einar addition on the fiber/matrix adhesion and consequently on the final mechanical properties of the composite. In particular, two analytical approaches were followed: one called “indirect” based on empirical parameters and one called “direct” that allows an IFSS evaluation. 

### 3.1. Indirect Approach

The first analytical approach is based on an evaluation of the fiber/matrix adhesion variation due to the Einar addition. The analytical models chosen contain empirical parameters whose variation is used as a yardstick to evaluate an improvement or worsening of the fiber/matrix adhesion caused by the Einar presence. The models that have been taken into account were the Pukánszky model [56] and the Sato and Furukawa model [57].

The composite strength varies with the fiber load, but also, the adhesion between the fibers and the polymeric matrix affects this mechanical property. For rigid fillers and for ultra-short fibers (as in this study), the filler reinforcing effect can be expressed by the following equation, proposed for the first time by Pukánszky [60]:

(1) $$\sigma_{c}=\;\sigma_{m}\frac{1-V_{f}}{1+2.5V_{f}}\exp\left(BV_{f}\right)$$

where *σ_c_* and *σ_m_* are the strength at break of the composite and matrix respectively, and *V_f_* is the volume fiber fraction. The term (1 – *V_f_*)/ (1 + 2.5*V_f_*) is correlated to a decrement of the effective load-bearing cross-section caused by the fibers’ addition, while the parameter B is an interaction parameter that considers the capacity of stress transmission between the matrix and the filler [61]. By writing Equation (1) in a linear form (Equation (2)), a linear correlation can be obtained in which the interaction parameter, B, is found as the slope of the Pukánszky’s plot (obtained plotting the natural logarithm of Pukánszky’s reduced strength, *σ_red_*, against the volume filler fraction):

(2) $$\ln\sigma_{red}=\ln\frac{\sigma_{c}\left(1+2.5V_{f}\right)}{\sigma_{m}\left(1-V_{f}\right)}=BV_{f}$$

Another possible evaluation of the fiber/matrix adhesion in short-fiber composites can be obtained on the basis of the values of the elastic moduli acquired experimentally and compared with the theoretical prediction. In this paper, in particular, the model developed by Sato and Furukawa was adopted (Equation (3) and Equation (4)). Also, this model contains parameters that depend on the matrix–filler interaction (*ξ*) and on shape distribution of the filler (*ψ*). Thanks to the Sato and Furukawa model, it is possible to correlate the tensile modulus of the composite with an adhesion parameter (*ξ*) that varies between 1 (indicating null adhesion) and 0 (perfect adhesion). The Sato and Furukawa equations are reported below:

(3) $$E=\;E_{m}\left[\left(1+\frac{0.5V_{f}^{\frac{2}{3}}}{1-V_{f}^{\frac{1}{3}}}\right)\right]\left(1-\psi \xi \right)-\frac{V_{f}^{\frac{2}{3}}\psi \xi }{\left(1-V_{f}^{\frac{1}{3}}\right)V_{f}}\;\;\;\;\;\;$$

(4) $$\psi =\left(\frac{V_{f}}{3}\right)\left(\frac{1+V_{f}^{\frac{1}{3}}-V_{f}^{\frac{2}{3}}}{1-V_{f}^{\frac{1}{3}}+V_{f}^{\frac{2}{3}}}\right)\;\;\;\;\;\;\;\;$$

where *E* and *E_m_* are the Young’s modulus of the composite and the matrix, respectively. 

### 3.2. Direct Approach

The second analytical approach is based on a quantitative evaluation of the fiber/matrix adhesion through the IFSS calculation. For this purpose, the Bader and Bowyer (B-B) model was considered. This model has been successfully adopted for various composite systems containing natural fibers [62,63,64,65,66,67,68]. 

The B-B model [53], discussed and analyzed by Thomason [54,69], is based on the Kelly-Tyson assumptions [70] for the IFSS prediction of a randomly reinforced polymeric composite system. The B-B model is an analytical iterative method that can be remarkably simplified for the case in which the fibers inside the composite are all below or above the critical length. The nomenclature adopted in the B-B equation is summarized in Table 3.

The B-B equation is written as follows (Equation (5)):

(5) $$\sigma_{c}=\eta_{0}(\underset{i}{\sum }\frac{\tau L_{i}V_{i}}{D}+\underset{j}{\sum }\left[E_{f}\varepsilon_{c}V_{j}\left(1-\frac{E_{f}\varepsilon_{c}D}{4\tau L_{j}}\right)\right])+\left(1-V_{f}\right)\sigma_{m}^{\prime }$$

Equation (5) can be rewritten in a simplified form (Equation (6)) as:

(6) $$\sigma_{c}=\eta_{0}\left(X+Y\right)+Z$$

where *X* is the stress contribution related to the fiber having a length below the critical fiber length, *Y* is the stress contribution related to the fibers above the critical length and *Z* is the stress matrix contribution. According to the Kelly-Tyson equation, the fibers shorter than the critical fiber length will carry an average stress equal to *τL/D*, while the fibers above the critical fiber length will carry a stress equal to $\;E_{f}\varepsilon_{c}\left(1-\frac{E_{f}\varepsilon_{c}D}{4\tau }\right)$. Generally, for short-fiber composites, a random orientation factor equal to 3/8 is supposed [71]; thus, with Equation (5), it is possible to find the *τ* value for which the theoretical stress at break calculated is equal to the experimental data. 

Recently [24], the B-B model was modified to better fit the experimental data for ultra-short fiber composite systems (like the composites studied in this work). It was found that for ultra-short fiber composite systems, all fibers are below the critical length and the expression of the B-B model (that in this case is simplified, Equation (7)) generates a great IFSS (τ) overestimation.

(7) $$\sigma_{c}=\eta_{0}\left[\frac{\tau LV_{f}}{D}+\left(1-V_{f}\right)\sigma_{m}^{\prime }\right]$$

This overestimation was attributed to the fiber ends effects that in long-fiber composites, involving a small portion of fiber, are negligible; nevertheless, for short- and ultra-short-fiber composites, these end effects cannot be neglected (they contribute significantly in the transferring of the load from the matrix to the fibers). Consequently, a modified B-B equation for the IFSS calculation containing the fiber ends stress contribution was found. The results obtained with the modified B-B equation for ultra-short fiber composites (reported in Equation (8)) [24], were thus compared with the classical B-B model.

(8) $$\sigma_{c}=\eta_{0}\left[V_{f}\left(\frac{\tau L}{D}+\sigma_{0}\right)+\left(1-V_{f}\right)\sigma_{m}^{\prime }\right]$$

In Equation (8), the fiber end stress contribution is named as *σ_0_*, and, in first approximation, it is retained constant and equal to the maximum stress value reached by the matrix alone [24].

## 4. Results and Discussion

**Figure 1:**
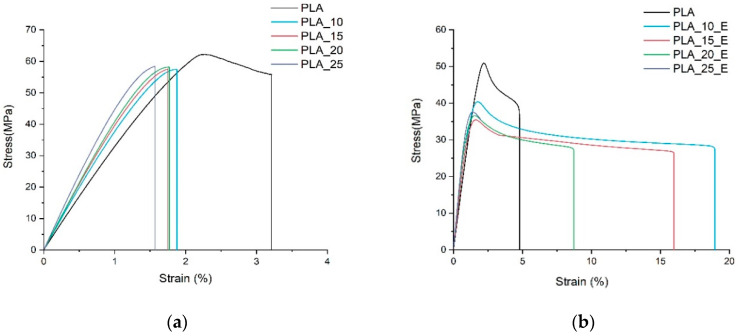
Stress-strain representative curves for pure PLA and for PLA/Arbocel fiber composites containing fibers content from 10 to 25 wt.%: (**a**) Composite system without additive, (**b**) composite system with the Einar addition.

**Figure 2:**
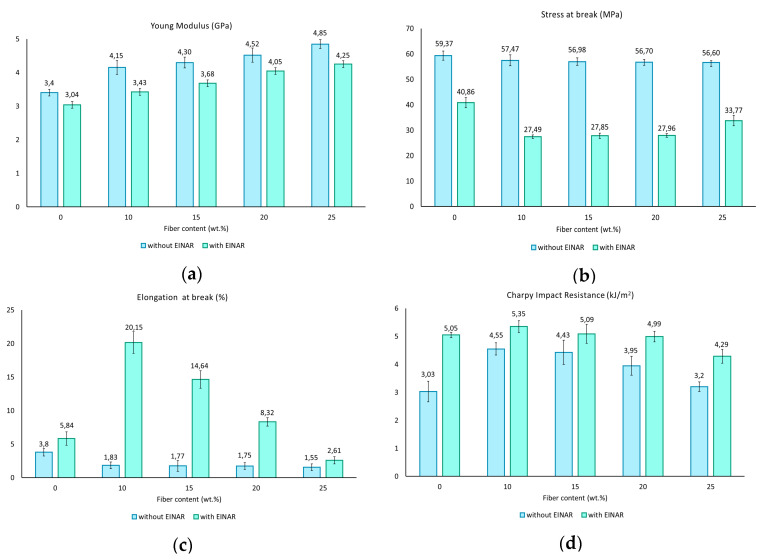
Mechanical properties for the two PLA/Arbocel composite systems: (**a**) Young modulus, (**b**) stress at break, (**c**) elongation at break, (**d**) Charpy impact resistance.

The results of the mechanical test are reported in Figure 1 and Figure 2. In particular, in Figure 1, the representative stress-strain curves obtained for the two composites systems studied are shown (with and without Einar), while in Figure 2, the main mechanical properties are summarized. PLA alone has the classical fragile break without yielding and with low strain at break. The addition of Einar makes PLA less fragile with a greater tendency for ductilization, and the elongation at break in fact increases at the expense of a stress at break and Young modulus decrements. The results of the Charpy impact test also confirms the PLA brittleness improvement (Charpy impact resistance passes from 3.03 to 5.05 kJ/m^2^ with Einar addition). The action of the Einar is similar to what happens when a plasticizer is added to PLA, where not only the processability but also the toughness is enhanced [23,72]. 

Regarding the Young modulus, both the two composites series trends (with and without Einar) are in agreement with those normally observed in other systems containing cellulosic fibers [22,29,73]. Since the fibers’ stiffness is several orders of magnitude higher than the matrix stiffness, it easily explains why by increasing the fibers amount, the final composite stiffness also increases. 

The trends of tensile stress and elongation at break are markedly different when Einar is added. In this case, the behavior is strongly affected by the fiber/matrix adhesion and fibers distribution. The behavior without Einar can be encountered in the literature for different composite systems [1,22,29,74]. In this case, considering the very low aspect ratio of the fibers, a weak but not bad adhesion it is expected due to the low decay of stress at break with fiber content amount. 

On the other hand, a decrease of the fiber/matrix adhesion can be expected with Einar. In this case, a marked decrement of the tensile stress is registered due to the impossibility of transferring the load from the matrix to the fibers in an efficient way. Nevertheless, this low adhesion is not negative; in fact, when the fibers have a very low aspect ratio, their behavior is similar to particulate-filled composites. In this case, if the filler is well dispersed in the matrix and if the adhesion with the matrix is low, the composite system could improve its toughness. In fact, according to the rigid filler toughening theory proposed by Argon and Cohen [75,76], the low adhesion of the filler with the matrix favors the debonding that can create voids, during tensile load, allowing the interparticle ligaments to deform plastically (absorbing energy). But, Einar, in this case, seems to have a double effect; in fact, by comparison to pure PLA, its addition makes the PLA matrix more ductile and this permits the final composite system to increase its toughness [50]. 

A low fiber/matrix adhesion improves not only the tensile toughness (defined as the area under the stress-strain curve), but also the impact resistance. A poor adhesion combined with a uniform filler dispersion does not allow an easy crack propagation, bringing about an impact resistance increment. 

Obviously, this positive toughening effect gradually tends to worsen as the fiber content increases. In fact, although the dispersing agent reduces the agglomerates’ formation, by increasing the fiber content, the resistant section of the composite decreases more and more; contemporarily, the probability to find defects and/or agglomerates increases. 

**Figure 3:**
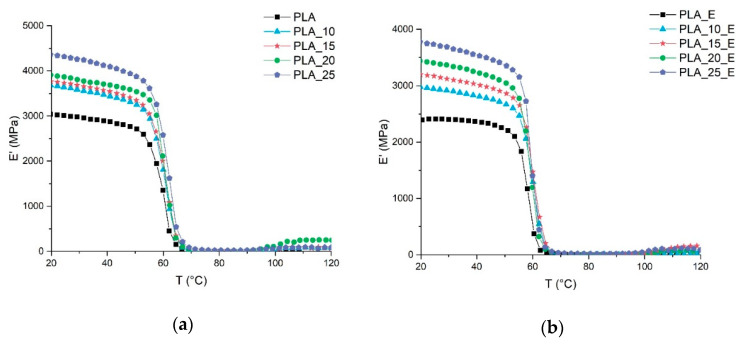
Storage modulus of PLA-Arbocel biocomposites as a function of temperature: (**a**) composite system without Einar, (**b**) composite system with Einar.

The results of DMTA, as shown in Figure 3, confirm the trends of mechanical tests. In fact, the influence of the fiber addition on the storage modulus (E’), as a function of temperature, increases with the fiber loading.

**Figure 4:**
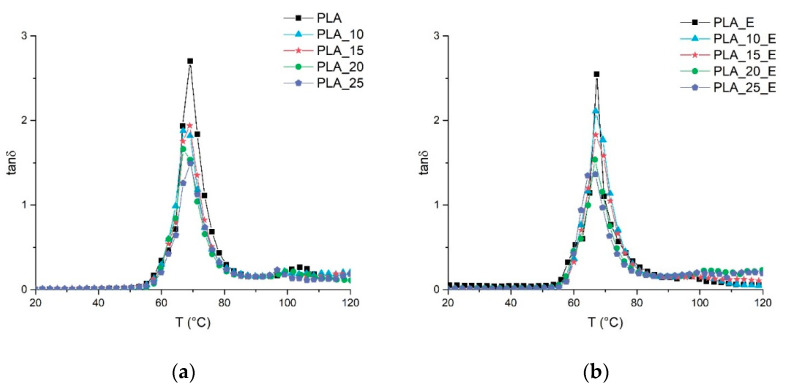
Tan δ of PLA-Arbocel biocomposites as a function of temperature: (**a**) composite system without Einar, (**b**) composite system with Einar.

From the plots of loss tangent (tan δ) (Figure 4) as a function of temperature, it can be observed that the fibers addition slightly shifts the temperature position of the tan δ peak and also slightly lowers the magnitude of the peak that becomes broad. The variation of the tan δ peak is generally correlated to the interaction between the fillers and the matrix that reduces the polymer chains’ mobility [77]. With the tan δ variation not marked, also, the matrix–filler interaction is not intense, also confirming the results of the mechanical tests.

It can also be observed that for all the formulations studied, the material will soften in correspondence of the PLA glass transition temperature (around 60 °C), which also causes a marked decay of the storage modulus. This decay is strictly correlated to the crystallinity of the material; in fact, the more crystalline the system, the less marked this decay will be. For all the systems studied, this decay is very marked, and it is correlated to the low tendency of the selected PLA grade to crystallize. The PLA used is basically amorphous. During the injection molding process, the mold must be heated in the PLA crystallization range (80–120 °C) if we want the PLA to start to crystallize [78]. In this study, the mold temperature was kept low (60 °C) so any eventual PLA crystallization phenomenon is avoided. Consequently, for all composite systems examined, a marked decay of the storage modulus is registered in correspondence of the glass transition temperature (tan *δ* peak). The ductilizing effect of Einar on PLA is similar, from the mechanical properties point of view, to that of the plasticizer, however, no PLA *T_g_* deviation (tan *δ* peak) can be encountered when Einar is added. 

Therefore, from the experimental mechanical data, it emerges that Einar diminishes the interaction between the fibers and the matrix. The following results obtained by the application of the analytical models are in agreement with experimental data. 

**Figure 5:**
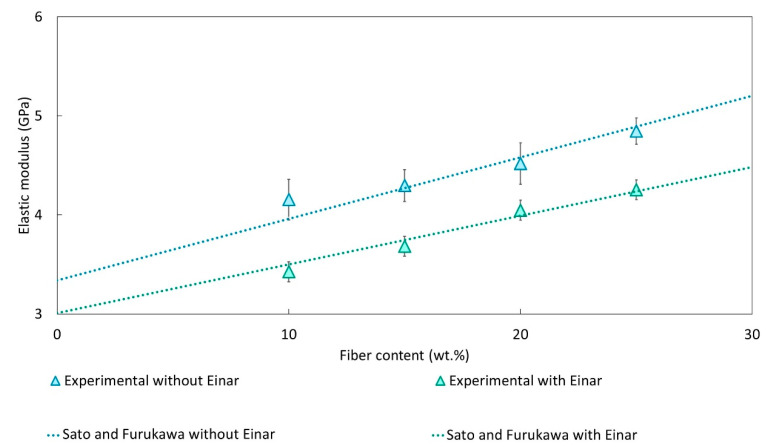
Comparison between the predicted elastic modulus obtained with the Sato and Furukawa model and the experimental data.

Table 4 reports the values of the adhesion parameter, ξ, obtained by the application of the Sato and Furukawa model, while the prediction of the elastic modulus compared with experimental data is reported in Figure 5. 

The ξ parameter was calculated by means of an iterative numerical method (using Excel Data Solver Function) on the basis of the experimental data obtained from the tensile test. A very good prediction between the predicted elastic modulus and the experimental data was found, confirming the applicability of this model to the systems studied. The adhesion parameter, for both composites systems examined, indicates that for the composites without Einar, the adhesion is not bad (the ξ parameter is close to 0). Nevertheless, the Einar addition provokes a marked adhesion decay, leading to an increment of ξ that passes from 0.13 to 0.37. 

However, it must be pointed out that the Sato and Furukawa model derive the adhesion parameter on the basis of the composites’ elastic modulus. But, it is well-known that the strength of the composite is more influenced by the fiber/matrix adhesion whose effect on composite ultimate tensile strength is larger than on stiffness [79]. Consequently, the Pukánszky’s B parameter variation gives a more precise variation of the fiber/matrix adhesion. 

**Figure 6:**
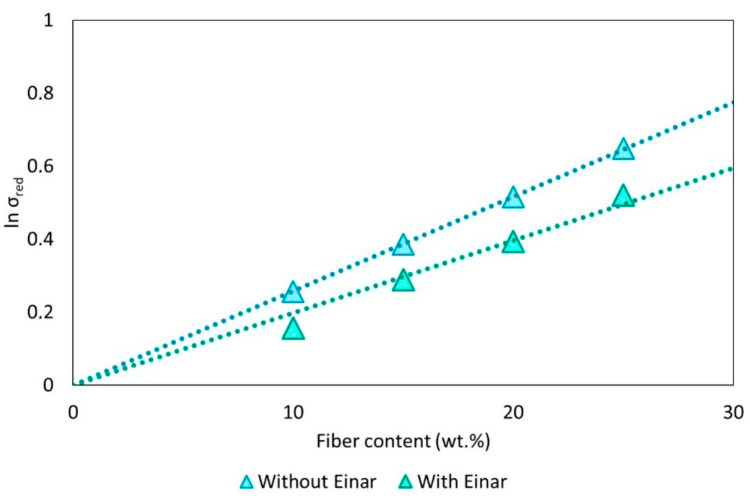
Pukánszky’s plot for PLA-Arbcocel composites with and without Einar.

The B parameter obtained from the Pukánszky’s plot (reported in Figure 6) is reported in Table 4.

It can also be observed that in this case, a decrease of the B value from 2.58 to 1.98 is registered, confirming a worsening of the matrix/filler interaction with Einar addition. 

The two models applied (Sato and Furukawa and Pukanszky models) confirmed the same tendency of Einar to decrease the fiber/matrix interaction. 

The quantitative evaluation, carried out by the IFSS calculation with the classical B-B model and its modified version, can be found in Table 5. With the ultra-short fiber, the non-iterative approach was adopted using Equations (7) and (8) for the classic and modified B-B models, respectively. The value of *σ*’*_m_* is the stress of the matrix at the composite elongation at break for the composite without Einar, in which the final elongation at break of the composite is inferior to the matrix elongation at break. While, for the system with Einar, in which the elongation at break of the matrix is inferior to that of composites, the final stress at break of the matrix was considered. An orientation factor equal to 3/8 valid for randomly oriented fiber composites was considered for both the systems analyzed. Because IFSS is an interface property that does not depend on the volumetric filler fraction [24,62,64,80], a mean value among the values obtained for the two composites systems at different volumetric percentages was considered and reported in Table 5. 

It can be observed that with the modified B-B model, the IFSS values are lower and more realistic. In fact, the IFSS values are too high with the classic B-B model, and for the system without Einar, the IFSS overcomes the Von Mises threshold. This threshold, generally adopted to discuss the fiber/matrix interfacial shear strength, is kept as a conservative value that cannot be exceeded. Thus, the maximum value that the shear strength reaches can be estimated using the von Mises relationship (*τ* = σ_m_/√3) [81].

On the other hand, a better estimation with the modified B-B model for the IFSS ultra-short fiber composites system is confirmed. Effectively, a marked decrease of the interfacial adhesion with the Einar addition can be confirmed. The system without Einar in fact shows a not bad adhesion with an IFSS value of 34.44 MPa that drops down to 8.20 MPa in the presence of Einar.

**Figure 7:**
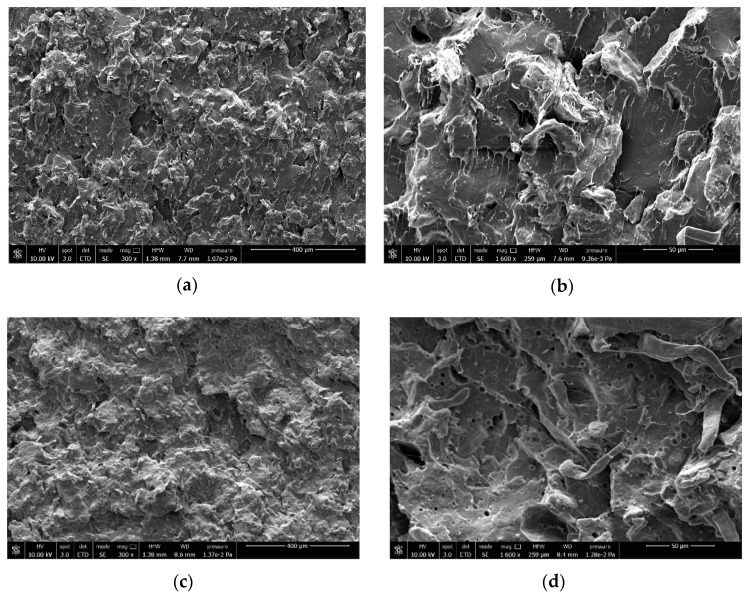

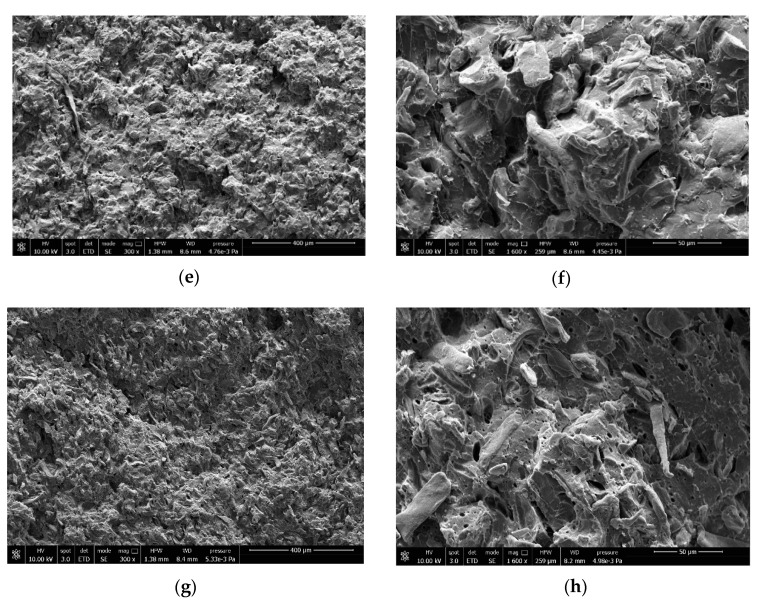
Scanning electron microscopy (SEM) images, at different magnification (300x and 1600x) of some of the composites investigated: (**a,b**) PLA_10, (**c**,**d**) PLA_10_E, (**e**,**f**) PLA_25, (**g**,**h**) PLA_25_E.

To get a complete view of the two composite systems studied, the morphology was investigated by SEM. Given the large number of images due to the different fiber amounts added, only the images (at 300× and 1600×) related to the minimum (10 wt.%) and maximum (25 wt.%) fiber content are reported in Figure 7. 

Thanks to the 300x magnification, it is possible to have an overall view of the composite morphology and in particular, it is possible to evaluate the effect of the Einar addition on the fibers’ dispersion inside the PLA matrix. Effectively, Einar favors a better dispersion of the cellulose fibers, reducing the agglomerates’ formation, and this effect is more evident where the fiber content is lower (at 10 wt.%). The images at higher magnification (1600×) allow a qualitative evaluation of the fiber/matrix adhesion. The SEM images confirm what emerged from the mechanical test and from the analytical models. The adhesion between the fibers and the matrix is lower when Einar is present. In fact, it can be observed that the fibers’ debonding is more pronounced for the composites containing Einar for which there is a greater quantity of fibers detached from the matrix. On the other hand, the composites without Einar remain better adhered to the matrix and a moderate pull-out is observed.

**Figure 8:**
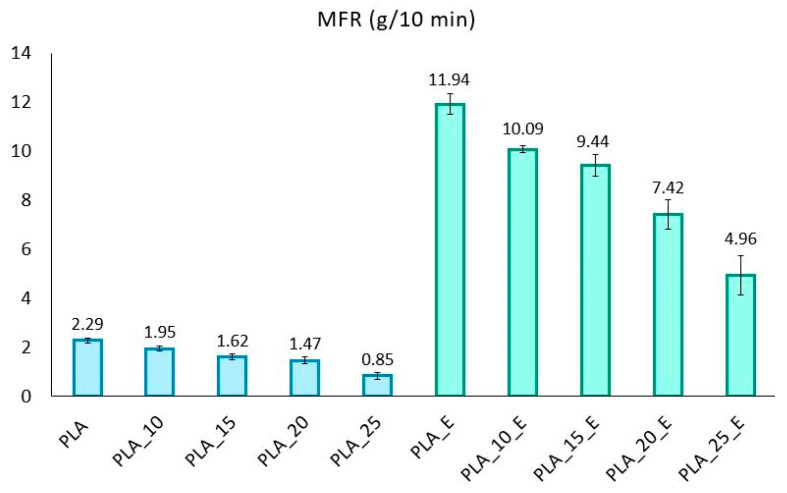
MFR values for PLA/Arbocel composites with and without Einar.

Finally, to conclude the composites’ characterization, it is of great interest, from a technical point of view, to know the fluidity of the molten material and the influence, therefore, of the filler and the dispersing aid on the MFR values and on the MVR along the time to predict the behavior of biocomposites during extrusion and injection molding. Figure 8 shows how the matrix fluidity is higher than for all biocomposites, showing how the fibers give strength to the melt in both cases (with and without Einar addition).

The viscosity increment with the fiber amount is proportionally similar in the two series of biocomposites, but, in absolute values, it is more evident in the series of materials with Einar in which it started from a value of about 12 for PLA with Einar to 4.96 when there is 25 wt.% of Arbocel.

The biocomposites with Einar are much more suitable for processes such as injection molding, where a high fluidity of the melt is required; indeed, in order to achieve a greater melt strength, the temperature profile of the injection molding of biocomposites with Einar has been lowered by 10 °C in each zone. In the absence of Einar, the values are those typical of processing operations where a greater melt strength is necessary. 

It is also interesting that the graph of the melt volume rate was measured in a time interval of 30 s, thus simulating a maximum residence time within a process [82]. 

**Figure 9:**
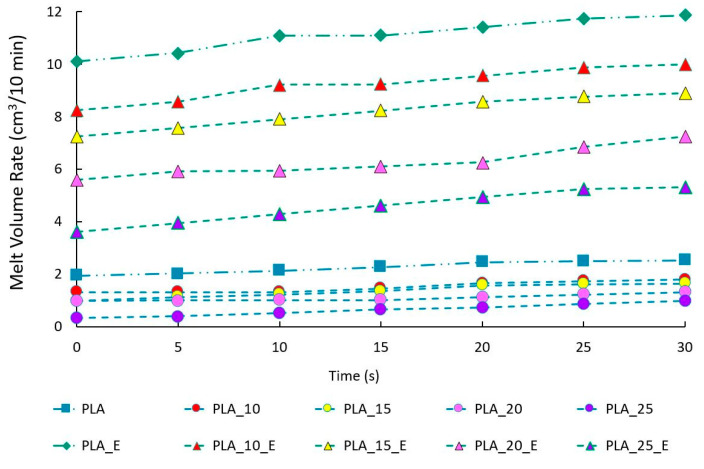
MVR trends for PLA/Arbocel composites with and without Einar.

Indeed, the duration that the polymer stays inside an extruder as a solid inside the solid bed is not important because a physical change such as mixing and chemical changes such as degradation or crosslinking do not occur in the solid polymer. The duration that the polymer stays inside an extruder as a physically and chemically active hot melt is the important residence time, and such duration will be called the "effective residence time". The residence time distribution depends on the flow pattern and mixing inside the screw channel. The results regarding the melt volume rate (Figure 9) showed a slight increase of the fluidity index with time, probably due to the triggering of degradative processes, however the range of growing is low, so it is fair to say that all the biocomposites studied in this paper are stable in the so-called "efficient residence time" of the work processes.

## 5. Conclusions

In this study, PLA/Arbocel fiber composites containing different fiber amounts (from 10 to 25 wt.%) were produced by extrusion and the injection molding technique. The composites were characterized in terms of rheological, morphological and thermo-mechanical properties. The adhesion between Arbocel fibers and PLA matrix was adequate and an embrittlement of the final composites was evidenced. To better disperse the fibers, a bio-based dispersing aid (Einar 101) was added; in this way, not only was the processing improved (an increase of the MFR was registered that enabled to add a higher quantity of fibers, maintaining an acceptable viscosity value) but so were the final mechanical properties. In fact, it was observed that the Einar addition led to a decrement of the interfacial adhesion between the fiber and the matrix. Furthermore, when Einar is added alone to the PLA matrix, it becomes more ductile. Consequently, thanks to both this fiber/matrix adhesion decrement and to the low aspect ratio of the fibers, combined with more PLA ductility achieved with the Einar addition, it was possible to obtain composites with tailored toughness (improved elongation at break and Charpy impact resistance).

The variation of the interfacial shear strength due to the Einar addition was also evaluated by adopting different analytical models and obtaining good fitting with experimental data. In particular, the quantitative evaluation of the IFSS was possible thanks to the application of the modified version of the Bader and Bowyer model, suitably created for ultra-short-fiber composites systems. 

## Figures and Tables

**Table 1:** Composites name and compositions.

Name	PLA-Arbocel wt.%	Einar 101 wt.%
PLA	100–0	-
PLA_10	90–10	-
PLA_15	85–15	-
PLA_20	80–20	-
PLA_25	75–25	-
PLA_E	100–0	5
PLA_10_E	90–10	5
PLA_15_E	85–15	5
PLA_20_E	80–20	5
PLA_25_E	75–25	5

**Table 2:** Injection molding conditions.

Main Injection Molding Parameters	Composites without Einar	Composites with Einar
Temperature profile from feeder to injection zone (°C)	180–185–180	170–175–170
Mold temperature (°C)	60	60
Injection holding time (s)	5	5
Cooling time (s)	25	25
Injection pressure (bar) ^1^	90–110	90–110

^1^ Increasing the fiber content, the injection pressure was incremented.

**Table 3:** Bader and Bowyer model nomenclature.

Symbol	Meaning
ε_c_	Composite strain
η_0_	Fiber orientation factor
σ_c_	Composite stress at break
σ*_f_	Fiber stress at break
σ’_m_	Stress of the matrix at ε_c_
E_f,m_	Elastic modulus of fiber (*f*) or matrix (*m*)
a_r_	Fiber aspect ratio
V_f_	Volume fraction of the fibers in the composite
τ	Interfacial Shear Strength (IFSS)
D	Average fiber diameter
L_c_	Critical fiber length
L_i,j_	Fiber length below (*i*) and above (*j*) the critical fiber length

**Table 4:** Values of the calculated parameter correlated to the fiber/matrix adhesion according to the Sato and Furukawa and Pukánszky models for the PLA/Arbocel composite system with and without Einar.

Composite System	Sato and Furukawa ξ Parameter	Pukánszky B Parameter
PLA/Arbocel	0.13	2.58
PLA/Arbocel/Einar	0.37	1.98

**Table 5:** IFSS evaluation for PLA/Arbocel composites with and without Einar.

Composite System	IFSS (MPa) According to Classic B-B Model (Equation (7))	IFSS (MPa) According to Modified B-B Model (Equation (8))	IFSS (MPa) Von Mises Threshold
PLA/Arbocel	55.92	34.44	37.20
PLA/Arbocel/Einar	23.20	8.20	25.98

## References

[1] Gurunathan T., Mohanty S., Nayak S.K. (2015). A review of the recent developments in biocomposites based on natural fibres and their application perspectives. Compos. Part A Appl. Sci. Manuf..

[2] La Mantia F.P., Morreale M. (2011). Green composites: A brief review. Compos. Part A Appl. Sci. Manuf..

[3] Mohanty A.K., Misra M., Drzal L.T. (2002). Sustainable bio-composites from renewable resources: opportunities and challenges in the green materials world. J. Polym. Environ..

[4] Râpă M., Popa M., Cinelli P., Lazzeri A., Burnichi R., Mitelut A., Grosu E. (2011). Biodegradable Alternative to Plastics for Agriculture Application. Rom. Biotechnol. Lett..

[5] Garrison T.F., Murawski A., Quirino R.L. (2016). Bio-Based Polymers with Potential for Biodegradability. Polymers (Basel).

[6] Righetti M.C., Cinelli P., Mallegni N., Massa C.A., Aliotta L., Lazzeri A. (2019). Thermal, Mechanical, Viscoelastic and Morphological Properties of Poly ( lactic acid ) based Biocomposites with Potato Pulp Powder Treated with Waxes. Materials (Basel).

[7] Dungani R., Karina M., Sulaeman A., Hermawan D., Hadiyane A. (2016). Agricultural waste fibers towards sustainability and advanced utilization: A review. Asian J. Plant Sci..

[8] Mohanty A.K., Vivekanandhan S., Pin J.-M., Misra M. (2018). Composites from renewable and sustainable resources: Challenges and innovations. Science.

[9] Bledzki A.K., Gassan J. (1999). Composites reinforced with cellulose based fibres. Prog. Polym. Sci..

[10] Satyanarayana K.G., Arizaga G.G.C., Wypych F. (2009). Biodegradable composites based on lignocellulosic fibers—An overview. Prog. Polym. Sci..

[11] Faruk O., Bledzki A.K., Fink H.P., Sain M. (2012). Biocomposites reinforced with natural fibers: 2000-2010. Prog. Polym. Sci..

[12] Pickering K.L., Efendy M.G.A., Le T.M. (2016). A review of recent developments in natural fibre composites and their mechanical performance. Compos. Part A Appl. Sci. Manuf..

[13] Raquez J.M., Habibi Y., Murariu M., Dubois P. (2013). Polylactide (PLA)-based nanocomposites. Prog. Polym. Sci..

[14] Aliotta L., Cinelli P., Coltelli M.B., Righetti M.C., Gazzano M., Lazzeri A. (2017). Effect of nucleating agents on crystallinity and properties of poly (lactic acid) (PLA). Eur. Polym. J..

[15] Scaffaro R., Lopresti F., Botta L. (2018). PLA based biocomposites reinforced with Posidonia oceanica leaves. Compos. Part B Eng..

[16] Aliotta L., Gigante V., Acucella O., Signori F., Lazzeri A. (2020). Thermal, Mechanical and Micromechanical Analysis of PLA/PBAT/POE-g-GMA Extruded Ternary Blends. Front. Mater..

[17] Gross R.A., Kalra B. (2002). Biodegradable Polymers for the Environment. Science.

[18] Scaffaro R., Botta L., Lopresti F., Maio A., Sutera F. (2017). Polysaccharide nanocrystals as fillers for PLA based nanocomposites. Cellulose.

[19] Chen Y., Geever L.M., Killion J.A., Lyons J.G., Higginbotham C.L., Devine D.M. (2016). Review of multifarious applications of poly (lactic acid). Polym. Plast. Technol. Eng..

[20] Nagarajan V., Mohanty A.K., Misra M. (2016). Perspective on Polylactic Acid (PLA) based Sustainable Materials for Durable Applications: Focus on Toughness and Heat Resistance. ACS Sustain. Chem. Eng..

[21] Barletta M., Aversa C., Puopolo M., Vesco S. (2020). Ultra-flexible PLA-based blends for the manufacturing of biodegradable tamper-evident screw caps by injection molding. J. Appl. Polym. Sci..

[22] Aliotta L., Gigante V., Coltelli M.B., Cinelli P., Lazzeri A. (2019). Evaluation of Mechanical and Interfacial Properties of Bio-Composites Based on Poly (Lactic Acid ) with Natural Cellulose Fibers. Int. J. Mol. Sci..

[23] Liu H., Zhang J. (2011). Research progress in toughening modification of poly(lactic acid). J. Polym. Sci. Part B Polym. Phys..

[24] Aliotta L., Lazzeri A. (2020). A proposal to modify the Kelly-Tyson equation to calculate the interfacial shear strength (IFSS ) of composites with low aspect ratio fibers. Compos. Sci. Technol..

[25] Rezaei F., Yunus R., Ibrahim N.A., Mahdi E.S. (2008). development of short-carbon-fiber-reinforced polypropylene composite for car bonnet Development of short-carbon-fiber-reinforced polypropylene composite for car bonnet. Polym. Plast. Technol. Eng..

[26] Tao Y.U., Yan L.I., Jie R.E.N. (2009). Preparation and properties of short natural fiber reinforced poly (lactic acid) composites. Trans. Nonferrous Met. Soc. China.

[27] Fu S.-Y., Lauke B., Mäder E., Yue C.-Y., Hu X. (2000). Tensile properties of short-glass-fiber-and short-carbon-fiber-reinforced polypropylene composites. Compos. Part A Appl. Sci. Manuf..

[28] Mohanty A.K., Misra M., Hinrichsen G. (2000). Biofibres, biodegradable polymers and biocomposites: An overview. Macromol. Mater. Eng..

[29] Aliotta L., Gigante V., Coltelli M.-B., Cinelli P., Lazzeri A., Seggiani M. (2019). Thermo-mechanical properties of PLA/short flax fiber biocomposites. Appl. Sci..

[30] Gigante V., Cinelli P., Righetti M.C., Sandroni M., Tognotti L., Seggiani M., Lazzeri A. (2020). Evaluation of Mussel Shells Powder as Reinforcement for PLA-Based Biocomposites. Int. J. Mol. Sci..

[31] Alimuzzaman S., Gong R.H., Akonda M. (2013). Nonwoven polylactic acid and flax biocomposites. Polym. Compos..

[32] Ibrahim N.A., Md Zin Wan Yunus W., Othman M., Abdan K., Hadithon K.A. (2010). Poly(Lactic Acid) (PLA)-reinforced kenaf bast fiber composites: The effect of triacetin. J. Reinf. Plast. Compos..

[33] Botta L., Fiore V., Scalici T., Valenza A., Scaffaro R. (2015). New polylactic acid composites reinforced with artichoke fibers. Materials (Basel).

[34] Mohanty A.K., Misra M., Drzal L.T. (2001). Surface modifications of natural fibers and performance of the resulting biocomposites: An overview. Compos. Interfaces.

[35] Mathew A.P., Oksman K., Sain M. (2005). Mechanical properties of biodegradable composites from poly lactic acid (PLA) and microcrystalline cellulose (MCC). J. Appl. Polym. Sci..

[36] Venkateshwaran N., Elaya Perumal A., Arunsundaranayagam D. (2013). Fiber surface treatment and its effect on mechanical and visco-elastic behaviour of banana/epoxy composite. Mater. Des..

[37] Sawpan M.A., Pickering K.L., Fernyhough A. (2011). Effect of fibre treatments on interfacial shear strength of hemp fibre reinforced polylactide and unsaturated polyester composites. Compos. Part A Appl. Sci. Manuf..

[38] Yan L., Chouw N., Yuan X. (2012). Improving the mechanical properties of natural fibre fabric reinforced epoxy composites by alkali treatment. J. Reinf. Plast. Compos..

[39] Baley C., Morvan C., Grohens Y. (2005). Influence of the absorbed water on the tensile strength of flax fibers. Proceedings of the Macromolecular Symposia.

[40] Abdelhak B., Noureddine M., Hacen M. (2018). Improvement of the interfacial adhesion between fiber and matrix. Mech. Mech. Eng..

[41] Valadez-Gonzalez A., Cervantes-Uc J.M., Olayo R., Herrera-Franco P.J. (1999). Effect of fiber surface treatment on the fiber–matrix bond strength of natural fiber reinforced composites. Compos. Part B Eng..

[42] Carman G.P., Reifsnider K.L. (1992). Micromechanics of short-fiber composites. Compos. Sci. Technol..

[43] Fu S.Y., Lauke B. (1996). Effects of fiber length and fiber orientation distributions on the tensile strength of short-fiber-reinforced polymers. Compos. Sci. Technol..

[44] Herrera-Franco P.J., Valadez-Gonzalez A. (2005). A study of the mechanical properties of short natural-fiber reinforced composites. Compos. Part B Eng..

[45] Abrate S. (1986). The mechanics of short-fiber-reinfroced composites: A review. Rubber Chem. Technol..

[46] Zainudin E.S., Sapuan S.M., Sulaiman S., Ahmad M.M.H.M. (2002). Fiber orientation of short fiber reinforced injection molded thermoplastic composites: A review. J. Inject. Molding Technol..

[47] Prashanth S., Subbaya K.M., Nithin K., Sachhidananda S. (2017). Fiber reinforced composites-a review. J. Mater. Sci. Eng..

[48] Capela C., Oliveira S.E., Pestana J., Ferreira J.A.M. (2017). Effect of fiber length on the mechanical properties of high dosage carbon reinforced. Procedia Struct. Integr..

[49] Bigg D.M. (1985). Effect of compounding on the properties of short fiber reinforced injection moldable thermoplastic composites. Polym. Compos..

[50] Aliotta L., Cinelli P., Coltelli M.B., Lazzeri A. (2019). Rigid filler toughening in PLA-Calcium Carbonate composites: Effect of particle surface treatment and matrix plasticization. Eur. Polym. J..

[51] Lazzeri A., Zebarjad S.M., Pracella M., Cavalier K., Rosa R. (2005). Filler toughening of plastics. Part 1—the effect of surface interactions on physico-mechanical properties and rheological behaviour of ultrafine CaCO3/HDPE nanocomposites. Polymer (Guildf.).

[52] Garg P., Singh B.P., Kumar G., Gupta T., Pandey I., Seth R.K., Tandon R.P., Mathur R.B. (2011). Effect of dispersion conditions on the mechanical properties of multi-walled carbon nanotubes based epoxy resin composites. J. Polym. Res..

[53] Bowyer W.H., Bader M.G. (1972). On the re-inforcement of thermoplastics by imperfectly aligned discontinuous fibres. J. Mater. Sci..

[54] Thomason J.L. (2002). Interfacial strength in thermoplastic composites-At last an industry friendly measurement method?. Compos. Part A Appl. Sci. Manuf..

[55] Tripathi D., Turton T., Chen F., Jones F.R. (1997). A new method to normalize the effect of matrix properties on the value of interfacial shear strength obtained from the fragmentation test. J. Mater. Sci..

[56] Pukánszky B. (1990). Influence of interface interaction on the ultimate tensile properties of polymer composites. Composites.

[57] Sato Y., Furukawa J. (1963). A molecular theory of filler reinforcement based upon the conception of internal deformation (a rough approximation of the internal deformation). Rubber Chem. Technol..

[58] (2011). International Organization for Standardization ISO 1133-1:2011: Determination of the melt mass-flow rate (MFR) and melt volume-flow rate (MVR) of thermoplastics - Part 1. Geneva.

[59] Lazzeri A., Phuong V.T. (2014). Dependence of the Pukánszky’s interaction parameter B on the interface shear strength (IFSS) of nanofiller- and short fiber-reinforced polymer composites. Compos. Sci. Technol..

[60] Huber T., Mussig J. (2008). Fibre matrix adhesion of natural fibres cotton, flax and hemp in polymeric matrices analyzed with the single fibre fragmentation test. Compos. Interfaces.

[61] Vallejos M.E., Espinach F.X., Julián F., Torres L., Vilaseca F., Mutjé P. (2012). Micromechanics of hemp strands in polypropylene composites. Compos. Sci. Technol..

[62] Li Y., Pickering K.L., Farrell R.L. (2009). Determination of interfacial shear strength of white rot fungi treated hemp fibre reinforced polypropylene. Compos. Sci. Technol..

[63] Gigante V., Aliotta L., Phuong V.T., Coltelli M.B., Cinelli P., Lazzeri A. (2017). Effects of waviness on fiber-length distribution and interfacial shear strength of natural fibers reinforced composites. Compos. Sci. Technol..

[64] del Rey R., Serrat R., Alba J., Perez I., Mutje P., Espinach F.X. (2017). Effect of sodium hydroxide treatments on the tensile strength and the interphase quality of hemp core fiber-reinforced polypropylene composites. Polymers (Basel).

[65] Andersons J., Modniks J., Joffe R., Madsen B., Nättinen K. (2016). Apparent interfacial shear strength of short-flax-fiber/starch acetate composites. Int. J. Adhes. Adhes..

[66] Raharjo W.P., Soenoko R., Purnowidodo A., Choiron M.A. (2018). Experimental and micromechanical modelling of randomly oriented zalacca fibre/low-density polyethylene composites fabricated by hot-pressing method. Cogent Eng..

[67] Modniks J., Poriķe E., Andersons J., Joffe R. (2012). Evaluation of the apparent interfacial shear strength in short-flax-fiber/PP composites. Mech. Compos. Mater..

[68] Thomason J.L. (2002). The influence of fibre length and concentration on the properties of glass fibre reinforced polypropylene: 5. Injection moulded long and short fibre PP. Compos. Part A Appl. Sci. Manuf..

[69] Kelly A., Tyson W.R. (1965). Tensile properties of fibre-reinforced metals: Copper/tungsten and copper/molybdenum. J. Mech. Phys. Solids.

[70] Sanadi A.R., Young R.A., Clemons C., Rowell R.M. (1994). Recycled Newspaper Fibers as Reinforcing Fillers in Thermoplastics: Part I-Analysis of Tensile and Impact Properties in Polypropylene. J. Reinf. Plast. Compos..

[71] Anderson K.S., Schreck K.M., Hillmyer M.A. (2008). Toughening Polylactide. Polym. Rev..

[72] Phuong V.T., Gigante V., Aliotta L., Coltelli M.B., Cinelli P., Lazzeri A. (2017). Reactively extruded ecocomposites based on poly(lactic acid)/bisphenol A polycarbonate blends reinforced with regenerated cellulose microfibers. Compos. Sci. Technol..

[73] Cinelli P., Mallegni N., Gigante V., Montanari A., Seggiani M., Coltelli B., Bronco S., Lazzeri A. (2019). Biocomposites Based on Polyhydroxyalkanoates and Natural Fibres from Renewable Byproducts. Appl. Food Biotechnol..

[74] Argon A.S., Bartczak Z., Cohen R.E., Muratoglu O.K. (2000). Novel mechanisms of toughening semi-crystalline polymers. Toughening of Plastics.

[75] Wilbrink M.W.L., Argon A.S., Cohen R.E., Weinberg M. (2001). Toughenability of Nylon-6 with CaCO3 filler particles: New findings and general principles. Polymer (Guildf.).

[76] Formela K., Hejna A., Piszczyk Ł., Saeb M.R., Colom X. (2016). Processing and structure–property relationships of natural rubber/wheat bran biocomposites. Cellulose.

[77] Aliotta L., Gazzano M., Lazzeri A., Righetti M.C. (2020). Constrained Amorphous Interphase in Poly (L -lactic acid): Estimation of the Tensile Elastic Modulus. ACS Omega.

[78] Cox H.L. (1952). The elasticity and strength of paper and other fibrous materials. Br. J. Appl. Phys..

[79] Serrano A., Espinach F.X., Julian F., Del Rey R., Mendez J.A., Mutje P. (2013). Estimation of the interfacial shears strength, orientation factor and mean equivalent intrinsic tensile strength in old newspaper fiber/polypropylene composites. Compos. Part B Eng..

[80] Pegoretti A., Della Volpe C., Detassis M., Migliaresi C., Wagner H.D. (1996). Thermomechanical behaviour of interfacial region in carbon fibre/epoxy composites. Compos. Part A Appl. Sci. Manuf..

[81] Shenoy A.V. (2013). Rheology of Filled Polymer Systems.

[82] Gasner G.E., Bigio D., Marks C., Magnus F., Kiehl C. (1999). A new approach to analyzing residence time and mixing in a co-rotating twin screw extruder. Polym. Eng. Sci..

